# Myocardial extracellular space volume in patients with systemic sarcoidosis: quantitative measurement using a T1 mapping technique

**DOI:** 10.1186/1532-429X-16-S1-P279

**Published:** 2014-01-16

**Authors:** Yuesong Yang, Anna Zavodni, Idan Roifman, Mohammad I Zia, Meyer Balter, Alexander Dick, Kim A Connelly, Graham A Wright

**Affiliations:** 1 Imaging Research and Schulich Heart Center, Sunnybrook Health Sciences Centre, University of Toronto, Toronto, Ontario, Canada; 2 Internal Medicine, Mount Sinai Hospital, University of Toronto, Toronto, Ontario, Canada; 3 Cardiology, Ottawa Heart Institute, Ottawa, Ontario, Canada; 4 Cardiology, St. Michael's, University of Toronto, Toronto, Ontario, Canada

## Background

Cardiac involvement is a major risk factor for sudden death in patients with systemic sarcoidosis. Pathologically, cardiac sarcoid lesions can be classified as early (mainly lymphocytic, similar to lymphocytic myocarditis), intermediate (active granulomatous), and late phase (primarily scar). These pathological processes can affect the myocardial extracellular space volume (ECV) in cardiac sarcoid. We hypothesize that quantitative measurement of ECV using a T1 mapping technique with CMR may yield new insights for the assessment of cardiac sarcoid.

## Methods

Nine patients with confirmed sarcoidosis were examined on a 1.5T MR system. First, a short-axis SSFP stack of slices covering the ventricles were acquired, and then a pre-contrast modified Look-Locker sequence was performed with 8 time points in a mid-LV level from which a T1 map and average LV myocardium T1 was derived. Post-contrast LGE-CMRs using IR-FGRE and multi-contrast late-enhancement were performed 10-15 minutes after a double-dose intravenous bolus injection of Gd-DTPA. A repeated T1 mapping sequence with the same localization as pre-contrast T1 measurement was acquired around 20 minutes post-contrast. The average myocardial T1 measurements did not exclude regions corresponding to LGE positive foci. LV function, LGE determination and quantitative T1 calculation used commercial software CMR42. Myocardial ECV was calculated according to the formula of ECV = (1-haematocrit) × (ΔR1myocardium/ΔR1blood).

## Results

**Figure 1:**
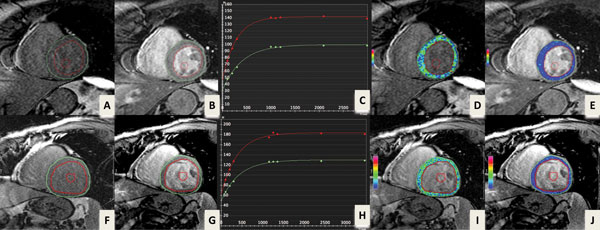
**Myocardial extracellular space volume (ECV) measurement using T1 mapping in sarcoid patients**. The upper row images (A-E) from a patient without cardiac involvement demonstrated a normal ECV of 25.3% and the lower row images (F-J) from a patient with cardiac involvement showed an increased ECV of 35.0%. A&F: one T1 raw image pre-contrast, B&G: one T1 raw image post-contrast, C&H: post-contrst T1 fitting curve (red line indicating blood pool and green line indicating myocardium), D&I: pre-contraset T1 map, E&J: post-contrast T1 map.

**Figure 2:**
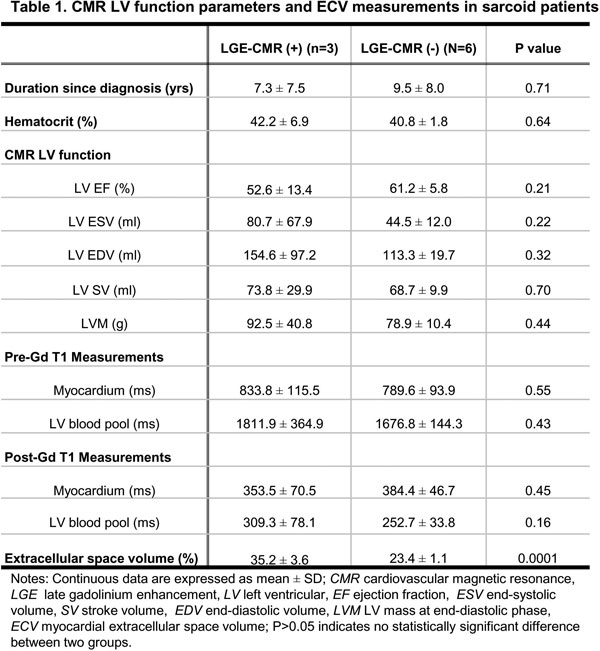
**CMR LV function parameters and ECV measurements in sarcoid patients**.

The LV functional parameters in nine subjects were in the normal range (LVEF = 58.3 ± 9.1%, LVESV = 56.6 ± 39.6 ml, LVEDV = 127.0 ± 55.0 ml, LVSV = 70.4 ± 17.0 ml, LVM = 83.5 ± 23.0 g). Three of nine subjects (33.3%) had cardiac involvement with LGE positive foci. Figure 1 illustrated T1 mapping in sarcoid patients (one with cardiac involvement and the other without). Figure 2 is a summary of LV function parameters, T1 and ECV measurements. There is no statistically significant difference in LV systolic functional parameters of LVEF, LVESV, LVEDV, LVSV and LVM between the LGE negative (n = 6) and LGE positive group (n = 3). The hematocrits and T1 measurements of myocardium and LV blood pool at pre- and post-contrast did not show a statistically significant difference between these two groups. However, a significantly increased ECV measurement (P = 0.0001) was observed in the LGE positive group (n = 3, 35.2 ± 3.6%) compared to the LGE negative group (n = 6, 23.4 ± 1.1%).

## Conclusions

This pilot study demonstrated increased myocardial ECV in patients with cardiac sarcoid, which may represent the combination of diffuse interstitial fibrosis and replacement scar. Further study is warranted for the clinical utility of T1 mapping and determination of ECV in the management of patients with cardiac sarcoid.

## Funding

NA.

